# Mice Deficient in Interferon-Gamma or Interferon-Gamma Receptor 1 Have Distinct Inflammatory Responses to Acute Viral Encephalomyelitis

**DOI:** 10.1371/journal.pone.0076412

**Published:** 2013-10-24

**Authors:** Eun-Young Lee, Kimberly L. W. Schultz, Diane E. Griffin

**Affiliations:** 1 W. Harry Feinstone Department of Molecular Microbiology and Immunology, Johns Hopkins Bloomberg School of Public Health, Baltimore, Maryland, United States of America; National Institute of Allergy and Infectious Diseases - Rocky Mountain Laboratories, United States of America

## Abstract

Interferon (IFN)-gamma is an important component of the immune response to viral infections that can have a role both in controlling virus replication and inducing inflammatory damage. To determine the role of IFN-gamma in fatal alphavirus encephalitis, we have compared the responses of wild type C57BL/6 (WTB6) mice with mice deficient in either IFN-gamma (GKO) or the alpha-chain of the IFN-gamma receptor (GRKO) after intranasal infection with a neuroadapted strain of sindbis virus. Mortalities of GKO and GRKO mice were similar to WTB6 mice. Both GKO and GRKO mice had delayed virus clearance from the brain and spinal cord, more infiltrating perforin^+^ cells and lower levels of tumor necrosis factor (TNF)-alpha and interleukin (IL)-6 mRNAs than WTB6 mice. However, inflammation was more intense in GRKO mice than WTB6 or GKO mice with more infiltrating CD3^+^ T cells, greater expression of major histocompatibility complex-II and higher levels of interleukin-17A mRNA. Fibroblasts from GRKO embryos did not develop an antiviral response after treatment with IFN-gamma, but showed increases in TNF-alpha, IL-6, CXCL9 and CXCL10 mRNAs although these increases developed more slowly and were less intense than those of WTB6 fibroblasts. These data indicate that both GKO and GRKO mice fail to develop an IFN-gamma-mediated antiviral response, but differ in regulation of the inflammatory response to infection. Therefore, GKO and GRKO cannot be considered equivalent when assessing the role of IFN-gamma in CNS viral infections.

## Introduction

Interferon (IFN)-gamma is an important component of the immune response to viral infections that can be produced by natural killer (NK), NKT and gamma/delta T cells early in infection and by CD4^+^ and CD8^+^ T cells later during the adaptive immune response. IFN-gamma can play an important role in protection, clearance and modulation of the immune response during infection [1–10]. However, prolonged expression of IFN-gamma in the central nervous system (CNS) can also lead to neuronal and glial cell damage [11–14]. 

Sindbis virus (SINV) is an arthropod-borne alphavirus of the family *Togaviridae* that also includes eastern equine encephalitis and western equine encephalitis viruses. SINV causes rash and arthritis in humans, but infects neurons in mice and provides a model system for study of the pathogenesis of encephalomyelitis. The severity of disease in infected adult mice is dependent on the viral strain and the genetic background of the mouse [15–17]. Relatively avirulent strains of SINV (e.g. AR339, TE) result in CNS infection from which the mice recover [18,19] and the process of virus clearance and recovery is dependent on local production of IFN-alpha/beta, antiviral antibody and IFN-gamma [3,20–22]. More virulent strains of SINV induce death from encephalomyelitis in susceptible strains of mice within 7-10 days after infection. NSV, a neuroadapted strain of SINV, was derived by passage of AR339 in mouse brain [23] and causes fatal encephalomyelitis after intracerebral or intranasal infection of C57BL/6 (B6) mice [16,17,24,25]. 

Virulence of NSV is associated with sequence changes in the 5’NTR and in the E1 and E2 glycoproteins that increase virus replication and cell death in mature neurons [15,26,27]. However, there is also a host contribution to fatal disease. The level of NSV replication is greater and spread within the CNS is faster in susceptible B6 mice than in resistant BALB/c mice [17]. This difference has been mapped to a quantitative trait locus on chromosome 2, but the responsible gene has not been identified [28]. The immune response to NSV infection in B6 mice leads to intense infiltration of mononuclear inflammatory cells [24,29,30] and outcome can be modulated by alteration of the immune response. Mice deficient in beta2 microglobulin, the major histocompatibility complex (MHC) light chain for class I and nonclassical MHC molecules, are protected from fatal NSV-induced encephalomyelitis [24,30]. Treatment with glutamate receptor antagonists also protects from paralysis and death in association with decreased CNS inflammation and delayed virus clearance [31,32]. 

To determine the role of IFN-gamma signaling in the pathogenesis of NSV-induced fatal encephalomyelitis, we have characterized mortality, virus distribution, replication and clearance, and the inflammatory response in IFN-gamma-/- (GKO) and IFN-gamma receptor 1-/- (GRKO) mice in comparison to wild type B6 (WTB6) mice. There were no differences in mortality, but virus clearance was delayed in both GKO and GRKO mice. Surprisingly, GKO and GRKO mice displayed marked differences in the quality and quantity of inflammatory cells entering the CNS in response to infection. GRKO mice had more infiltrating CD3^+^ T cells, more MHC class II expression and higher levels of IL-17 mRNA than WTB6 or GKO mice suggesting residual ability to regulate genes important for inflammation.

## Results

### Mortality

**Figure 1:**
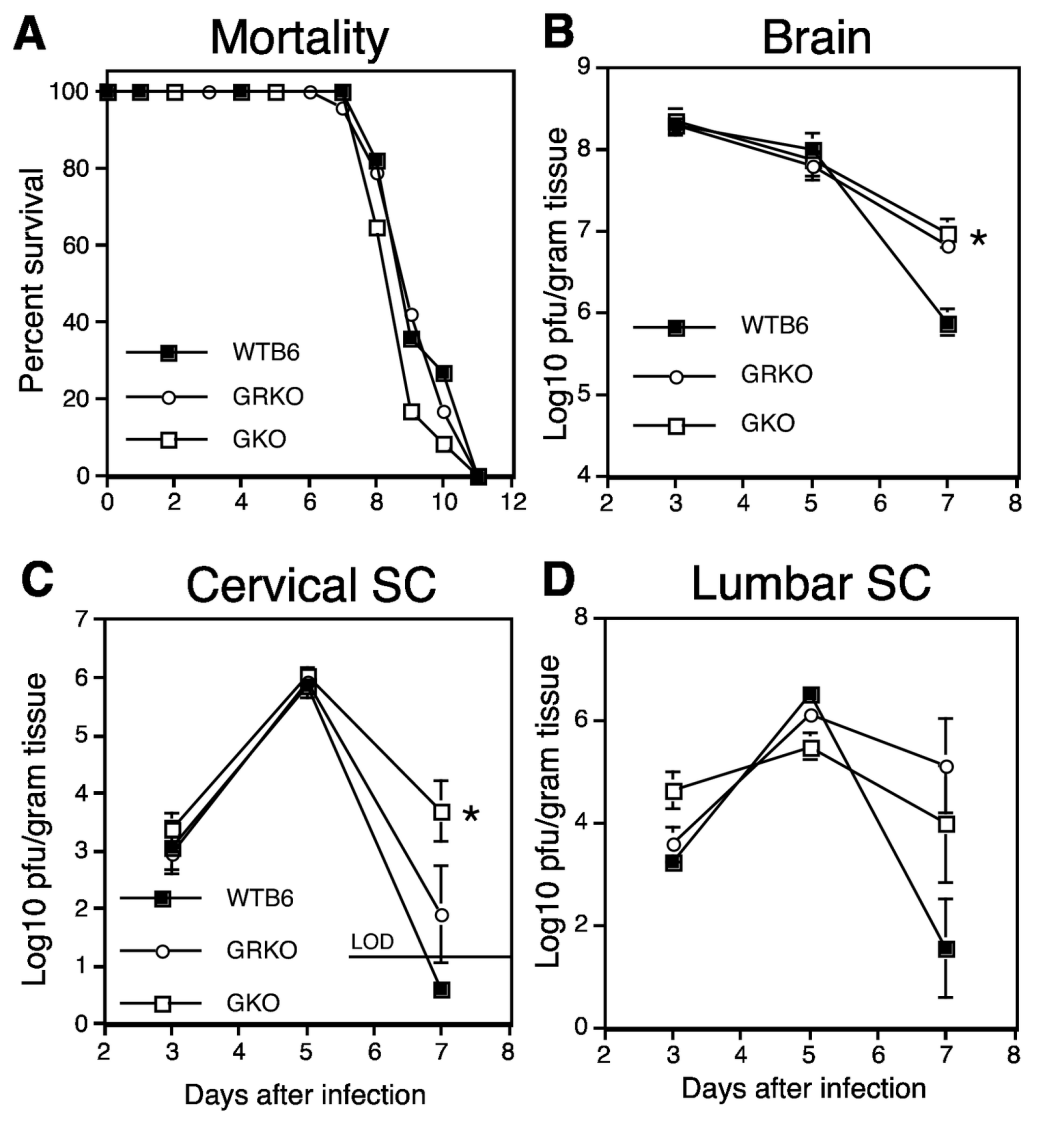
Mortality and virus replication after intranasal NSV infection. (A) 6-9 week-old WTB6 (*N* = 11), GKO (*N* = 24) and GRKO (*N* = 23) mice were infected intranasally with 10^5^ pfu NSV and followed daily for mortality which was similar. *P* = 0.47. At 3, 5, and 7 d after infection, brains (B), cervical (C) and lumbar (D) spinal cords from 3 WTB6, GKO or GRKO mice were assayed for infectious virus by plaque formation on BHK cells. LOD = limit of detection. * *P* < 0.02; t test.

To determine whether deficiencies of IFN-gamma or IFN-gammaR1 affected the outcome of NSV-induced encephalomyelitis, animals were followed for clinical disease. Mice in all groups showed weakness, paralysis and weight loss beginning 5-6 days after infection and died 8-11 days after infection. Mean time to death did not differ significantly between WTB6 (9.5 days), GRKO (9.3 days) and GKO (8.9 days) mice (Figure 1A) (*P* = 0.48).

### NSV replication and localization in the brain

To determine the effect of lack of IFN-gamma and IFN-gammaR1 on virus replication and clearance of infectious virus, brain and cervical and lumbar spinal cord homogenates were analyzed by plaque assay (Figure 1B-D). At 3 and 5 days after infection, the amounts of virus in CNS tissues were similar. However, at 7 days after infection NSV titers were lower in the brains (Figure 1 B; P < 0.02) of WTB6 than GRKO and GKO mice. Spinal cord titers were more variable, but all (N=3) WTB6 mice had cleared virus from the cervical cord (Figure 1C; P = 0.02) and 2 of 3 WTB6 mice had cleared virus from the lumbar spinal cord (Figure 1D) on day 7 while infectious virus was still present in 10 of 12 GKO and GRKO mice. 

**Figure 2:**
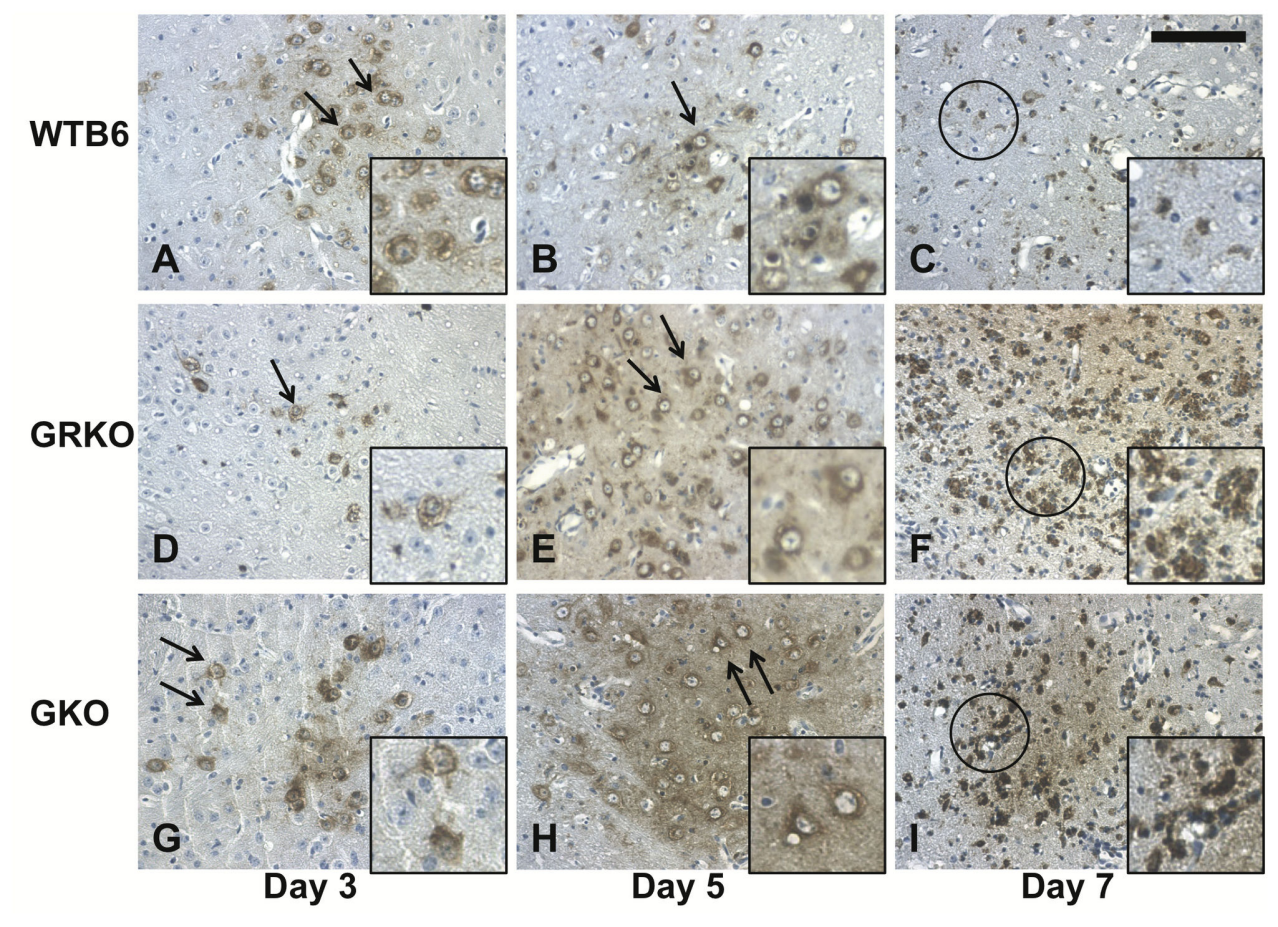
Replication and spread of virus in the brain after intranasal infection with NSV. Representative thalamus sections from the brains of at least 3 WTB6, GRKO and GKO mice 3, 5 and 7 days after intranasal infection with 10^5^ pfu NSV were stained with rabbit polyclonal antibody to NSV followed by biotinylated antibody to rabbit IgG, avidin-peroxidase and DAB. NSV (brown staining) was observed in neurons (arrows) at 3 (A, D, G) and 5 (B, E, H) days after infection in all strains of mice. At 7 days after infection NSV antigen was decreased in WTB6 (C), but not GRKO (F) or GKO (I) mice and neurons (circled) were degenerating. Insets show 2-fold magnified images of the NSV-positive neurons indicated by the arrows or circles. X300. Scale bar = 100um.

After intranasal infection, NSV is first detected in olfactory bulb neurons [17]. By 3 days after infection, NSV-infected neurons were observed in the pyriform cortex, amygdala, and septal area. By day 3-5, virus had spread to the hippocampus, cerebral cortex, hypothalamus and thalamus (Figure 2), and eventually to the cerebellum and spinal cord. The pathways utilized and time course of virus spread were similar in all groups of mice. Viral antigen was localized within neurons at days 3 (Figure 2A,D,G) and 5 (Figure 2B,E,H). At 7 days after infection, the amount of viral antigen was decreasing in WTB6 (Figure 2C), but not in GRKO (Figure 2F) or GKO (Figure 2I) mice and infected cells had lost their neuronal characteristics.

**Figure 3:**
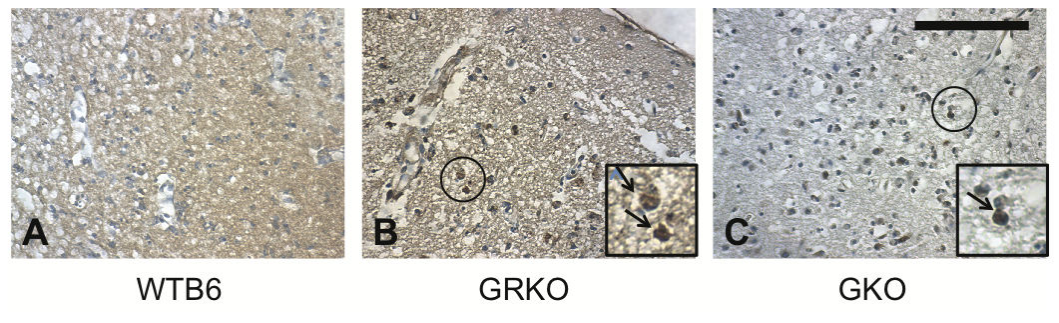
Immunohistochemical staining for nonstructural protein 3 in the piriform cortex. Brains from WTB6 (A), GRKO (B), and GKO (C) mice were examined 7 days after intranasal infection with 10^5^ pfu NSV. Representative sections from at least 3 mice were stained with a rabbit polyclonal antibody to nsP3 followed by biotinylated antibody to rabbit IgG, avidin-peroxidase and DAB. nsP3-positive cells were more frequent in GRKO and GKO mice. Insets contain magnified versions of encircled cells. X400. Scale bar = 100 um.

To further document continued virus replication in GKO and GRKO mice, sections of brain from 7 days after infection were stained for nsP3, a nonstructural viral protein synthesized during active replication (Figure 3). nsP3 was more abundant in the neurons of GRKO and GKO mice than in WTB6 mice.

### Effect of IFN-gamma on signaling, gene induction and NSV replication in WTB6 and GRKO mouse embryo fibroblasts (MEFs)

**Figure 4:**
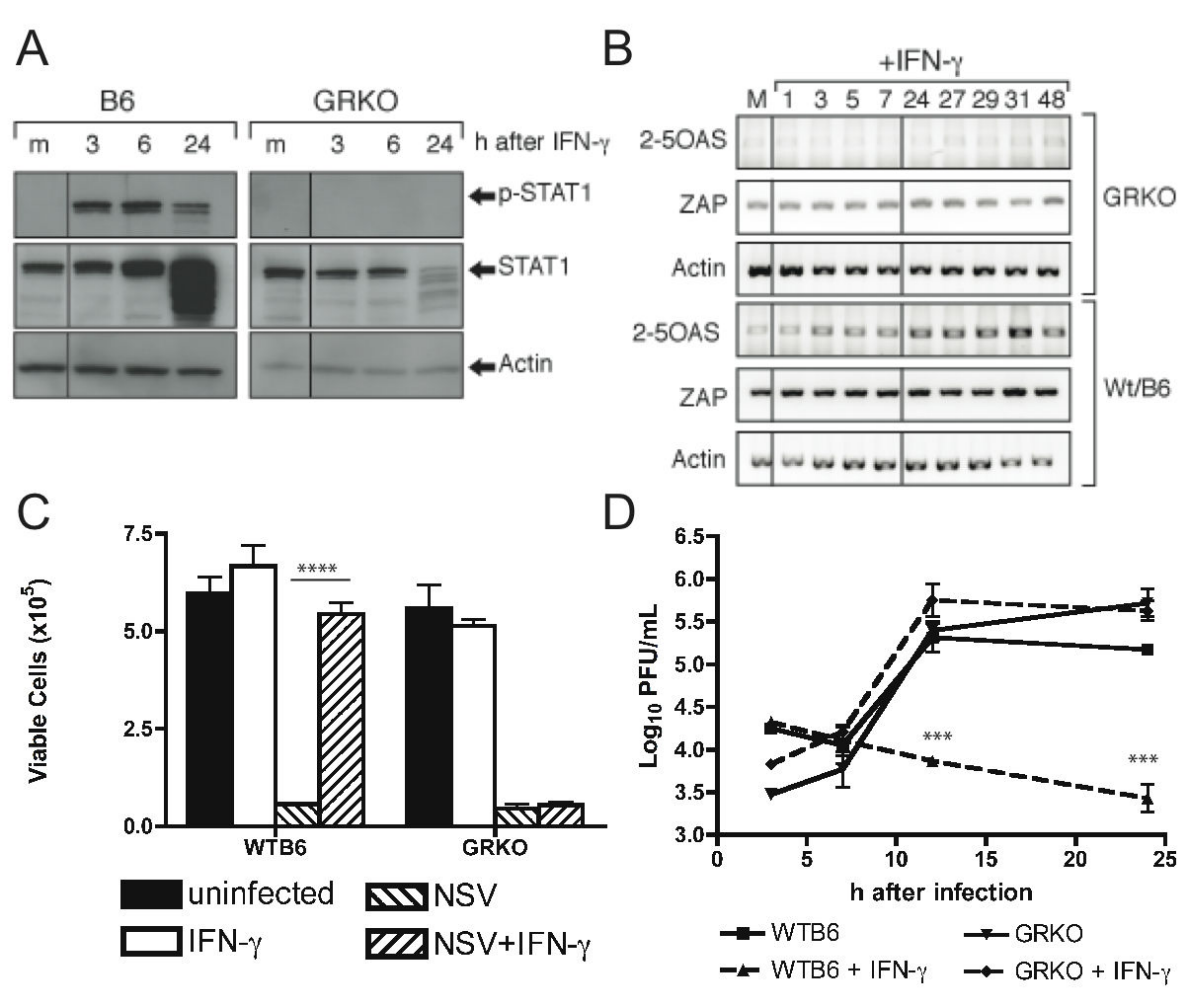
Effect of IFN-gamma on NSV infection of mouse embryo fibroblasts from GRKO and WTB6 mice. The responses of MEFs from WTB6 and GRKO mice to treatment with 100 U recombinant rat IFN-gamma were assessed over 24-48h. (A) Immunoblot to detect phosphorylation of STAT1 tyrosine 701 and changes in STAT1 protein levels. A representative of 3 experiments is shown. (B) RT-PCR to detect changes in transcription of antiviral genes 2-5 oligoadenylate synthase (OAS) and zinc finger antiviral protein (ZAP). Data shown are representative of 3 experiments. (C) Cell viability as determined by trypan blue exclusion 24h after infection with NSV (MOI = 5) in the presence and absence of IFN-gamma. Data are averaged from 2 experiments and plotted as mean +/- SEM. **** P < 0.0001; ANOVA (D) Effect of IFN-gamma on production of infectious virus by WTB6 (squares) and GRKO (triangles) MEFs after NSV infection with (dashed line) and without (solid line) IFN-gamma pretreatment. Data are from three experiments and plotted as mean +/- SEM. *** P < 0.001; ANOVA.

Canonical IFN-gamma signaling functions through phosphorylation of STAT-1 by the Jak kinases associated with the IFN-gamma receptor alpha-chain. To identify potential antiviral responses of GRKO cells to IFN-gamma, STAT phosphorylation and mRNA induction were assessed (Figure 4). IFN-gamma induced phosphorylation and upregulation of STAT-1 in WTB6, but not GRKO MEFs within 3h (Figure 4A) and GRKO MEFs showed no increase in expression of mRNA for the IFN-stimulated antiviral gene 2,5 oligoadenylate synthase (OAS) (Figure 4B). Zinc finger antiviral protein (ZAP), a PARP family member protein that restricts alphavirus replication [33], was constitutively expressed in both GRKO and WTB6 MEFs, but at lower levels in GRKO cells (Figure 4B). Pretreatment with IFN-gamma did not protect GRKO MEFs from NSV infection (Figure 4D) or virus-induced cell death (Figure 4C), while WTB6 MEFs were protected from both.

### Identification and quantification of inflammatory cells

**Figure 5:**
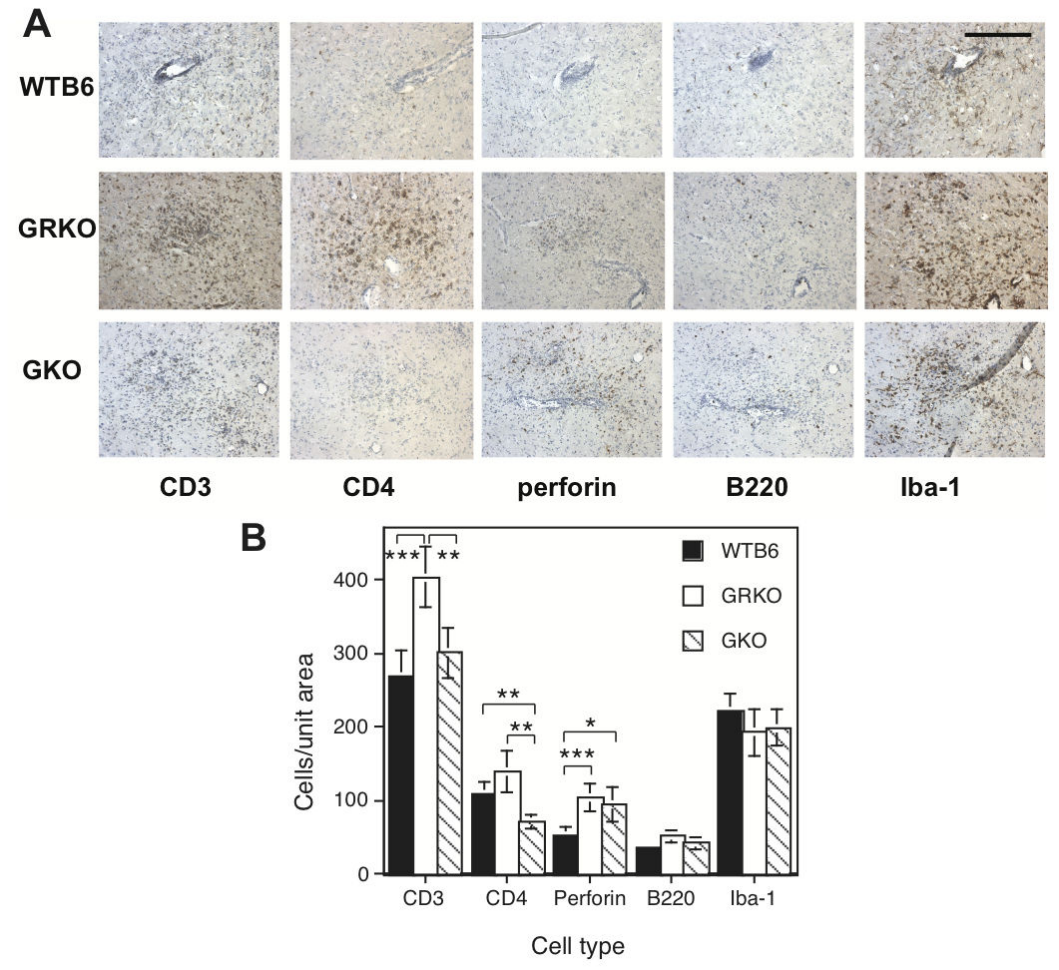
Immunohistochemical staining and quantification of inflammatory cells in the thalamus. Brains from WTB6, GRKO and GKO mice 7 days after intranasal infection with 10^5^ pfu NSV were examined for CD3^+^, CD4^+^, perforin^+^, B220^+^ and Iba-1^+^ cells. Frozen sections of the thalamus from 2 animals per group were immunostained with rabbit polyclonal anti-CD3, rabbit polyclonal anti-CD4, rat monoclonal anti perforin, rat monoclonal anti-B220 and rabbit polyclonal anti Iba-1. (A) Immunohistochemical staining for inflammatory cells. Representative sections are shown. X200. Scale bar = 200 um. (B) Photographs of 13-16 unit areas (0.25 mm^2^)/group were taken (X200 magnification) and numbers of brown stained immune cells were counted. Each bar indicates the mean and SEM. * *P* < 0.05, ** *P* < 0.01, *** *P* < 0.001; t test.

Inflammatory cells were evaluated by immunohistochemical staining. Mononuclear cell infiltration into the CNS was easily detectable 5 days after infection and increased at 7 days. CD3^+^, CD4^+^, perforin^+^, B220^+^ and Iba^+^ cells were present in areas of inflammation in all three groups of mice (Figure 5A), but the numbers and relative proportions of these cells differed (Figure 5B). CD3^+^ T cells were more abundant in GRKO mice than WTB6 (*P* < 0.001) or GKO (*P* < 0.01) mice. The numbers of perforin^+^ cells were higher in both GRKO (*P* < 0.01) and GKO (*P* < 0.02) mice than in WTB6 mice. However, there were fewer CD4^+^ T cells infiltrating the brains of GKO mice than GRKO or WTB6 mice (*P* < 0.01). Numbers of B220^+^ B cells and Iba-1^+^ microglial cells were similar. Therefore, IFN-gammaR1 deficiency resulted in infiltration of more perforin^+^ cells and CD3^+^ T cells into the CNS compared with WTB6 mice, while a lack of IFN-gamma resulted in infiltration of more perforin^+^ cells, a similar number of CD3^+^ T cells, but fewer CD4^+^ T cells than WTB6 mice.

### MHC class I and class II expression

**Figure 6:**
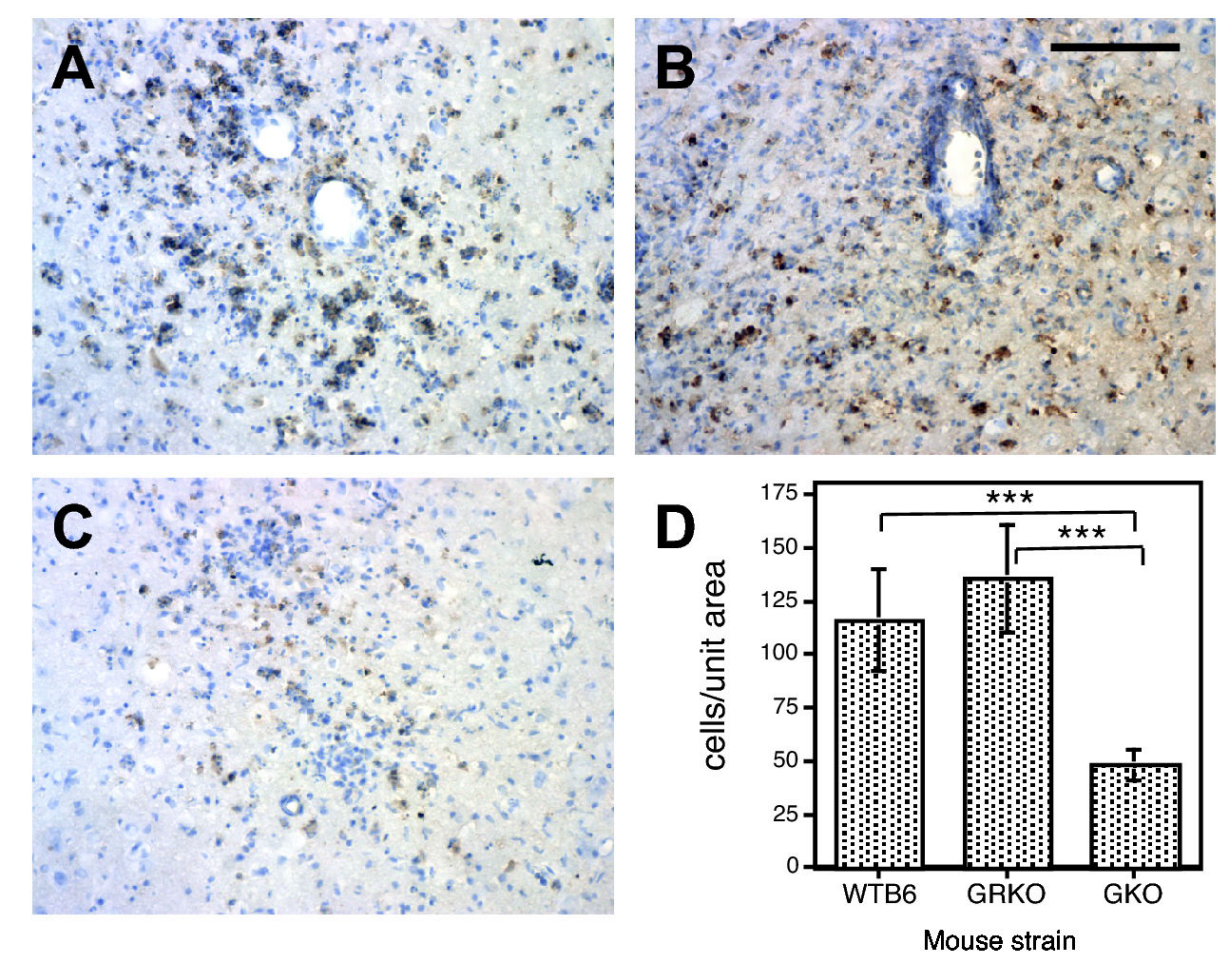
Immunohistochemical staining for MHC class I. Frozen sections from the thalamus of WTB6 (A), GRKO (B) and GKO (C) mice were stained with mouse monoclonal anti-H-2K^b^/H-2D^d^ for identification of MHC class I antigen-positive cells 7 days after intranasal infection with 10^5^ pfu NSV. Representative sections are shown. X250. Scale bar = 100 um. (D) Quantification of MHC class I-positive cells/0.16 mm^2^ (unit area) in thalamus tissue from 2 mice in each group. Photomicrographs of 5-7 areas were taken (250X magnification) and numbers of stained cells were counted. Each bar indicates the mean +/- SEM. *** P < 0.001; t test.

**Figure 7:**
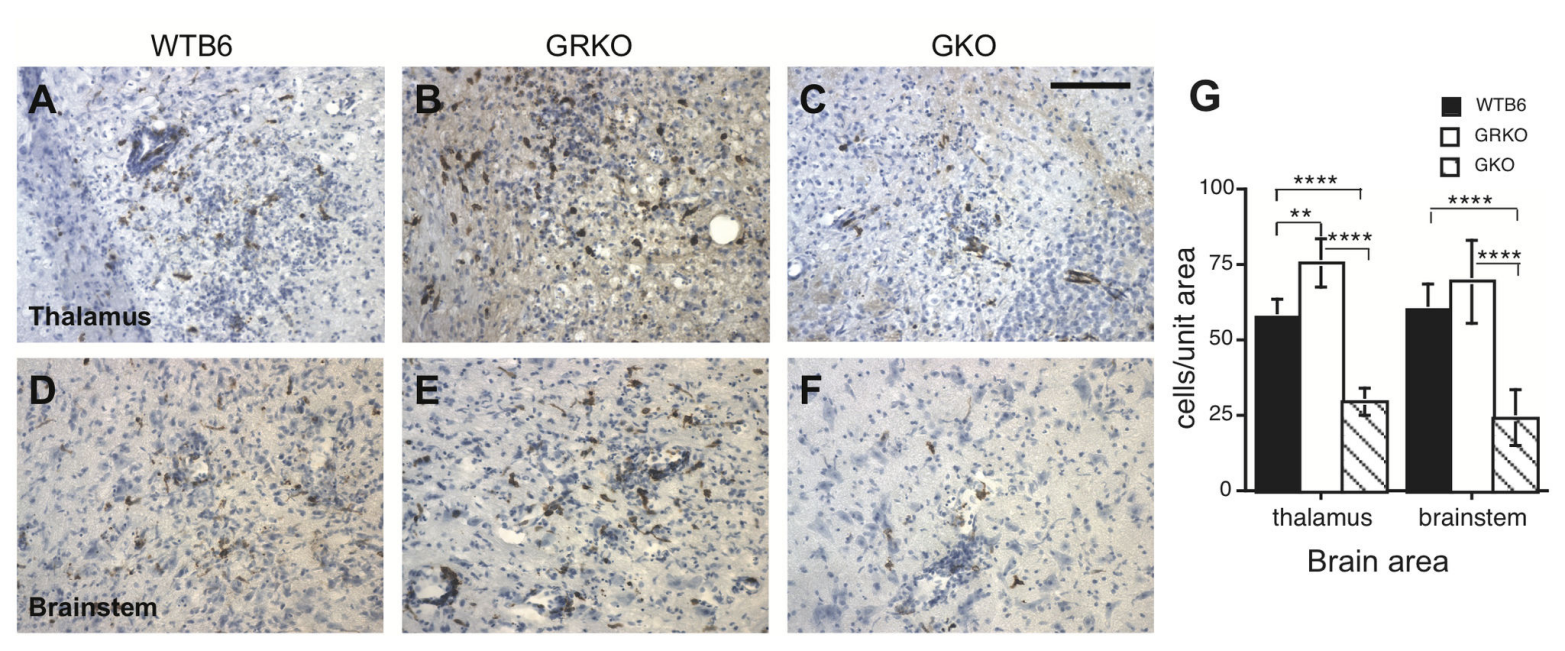
Immunohistochemical staining for MHC class II. Frozen sections from the thalamus (A-C, upper panels) and brainstem (D-F, lower panels) of WTB6 (A,D), GRKO (B,E) and GKO (C,F) mice were stained with mouse monoclonal anti-I-A/I-E for identification of MHC class II antigen-positive cells 7 days after intranasal infection with 10^5^ pfu NSV. X250. Scale bar = 100 um. (G) Quantification of MHC class II-positive cells/0.16 mm^2^ tissue in thalamus and brainstem from 2 animals in each group. Photomicrographs of 5-6 areas of thalamus and 10 areas of brainstem were taken and stained cells counted. Each bar indicates the mean and SEM. ** P < 0.01, *** P < 0.001, **** P < 0.0001; t test.

MHC-I and MHC-II immunoreactive cells were present in areas of inflammation of all NSV-infected mice at 7 days after infection when neurologic signs were present and inflammation was greatest (Figures 6 and 7). MHC-I-positive cells were more numerous in WTB6 (*P* = 0.0006) and GRKO (*P* = 0.0003) mice than in GKO mice (Figure 6). MHC-II-positive cells were round, oval or slender in shape consistent with round microglia and were present in inflamed regions of the parenchyma of the thalamus (Figure 7A-C), brainstem (Figure 7D-F), cerebral cortex, hippocampus and in the walls of inflamed blood vessels. MHC class II-positive cells were more numerous in the thalamus of GRKO mice than WTB6 mice (*P* = 0.0033) and were less numerous in the thalamus and brainstem of GKO mice than GRKO (*P* < 0.0001) or WTB6 (*P* < 0.0001) mice (Figure 7G).

### Measurement of cytokine mRNAs

**Figure 8:**
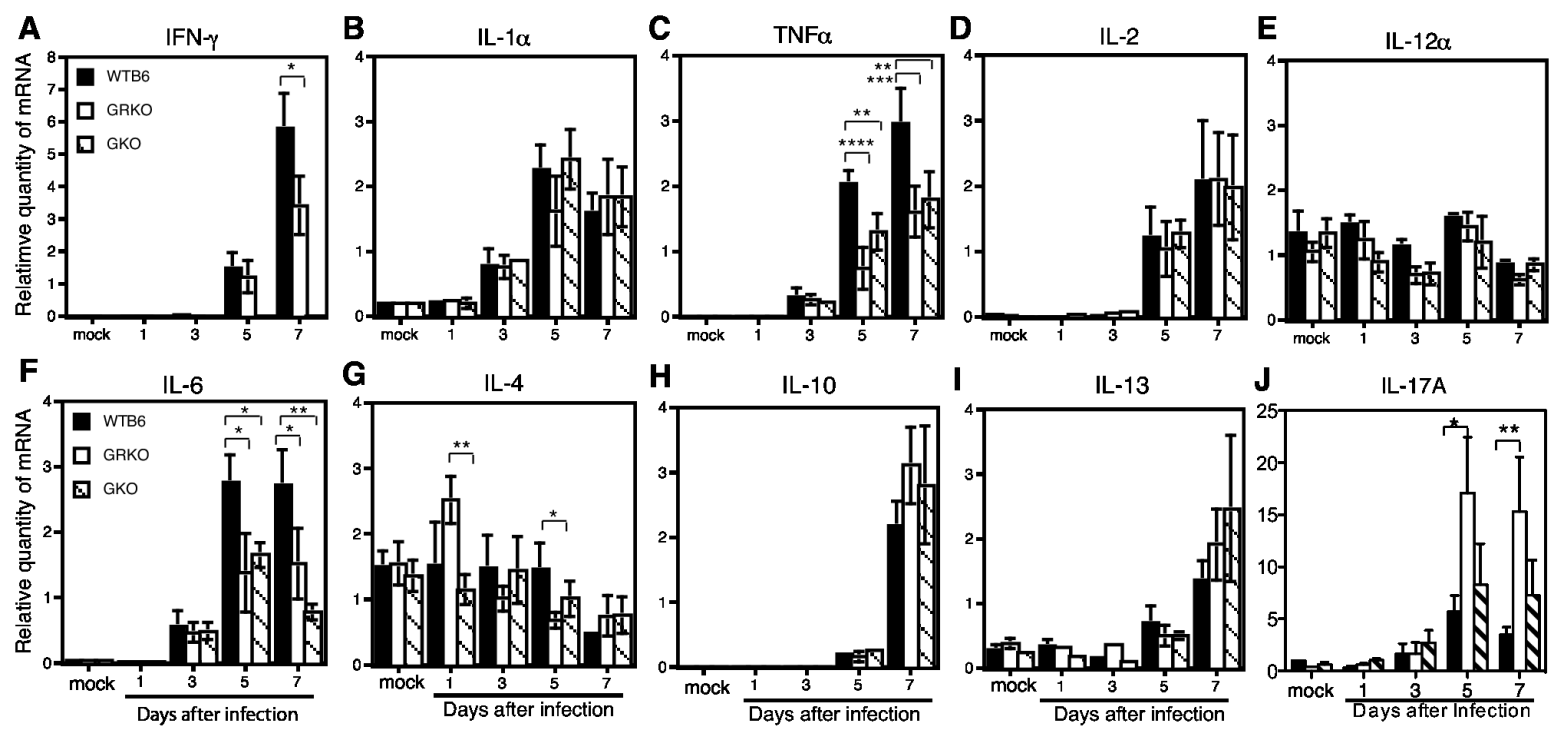
Quantification of cytokine mRNA levels in the brain. Quantitative RT-PCR was used to measure the levels of IFN-gamma (A), IL-1alpha (B), TNF-alpha (C), IL-2 (D), IL-12alpha (E), IL-6 (F), IL-4 (G), IL-10 (H), IL-13 (I) and IL-17A (J) mRNAs in the brains of uninfected (mock) and NSV-infected WTB6, GKO and GRKO mice. Cytokine mRNAs were normalized to beta-actin mRNA. Data are from 3 animals from each group at each time point and 2-4 experimental replicates. Bars indicate the mean and SEM for each group. * *P* < 0.05, ** *P* < 0.01, *** *P* < 0.001, **** *P* < 0.0001; ANOVA.

To determine if the differences in inflammatory responses were associated with differences in production of other cytokines and to verify the phenotype of GKO mice, IFN-gamma, IL-1alpha, TNF-alpha, IL-6, IL-12alpha, IL-2, IL-4, IL-10, IL-13 and IL-17A mRNAs were measured by qRT-PCR (Figure 8). IFN-gamma mRNA levels increased by day 5 and at day 7 were higher in WTB6 than GRKO mice (Figure 8A; P = 0.0103). As expected, IFN-gamma mRNA was undetectable in GKO mice. Increases in TNF-alpha and IL-6 mRNAs were detected by day 3 and were substantially higher in WTB6 mice than GRKO and GKO mice on days 5 and 7 (Figure 8C, F). IL-17A mRNA was increased at days 5 and 7 with higher levels in GRKO mice (Figure 8J). IL-1alpha, IL-2, IL-10 and IL-13 mRNA levels increased similarly during infection in all groups (Figure 8B,D,H,I). There was little change in IL-12 or IL-4 mRNA levels during infection (Figure 8E,G). 

**Figure 9:**
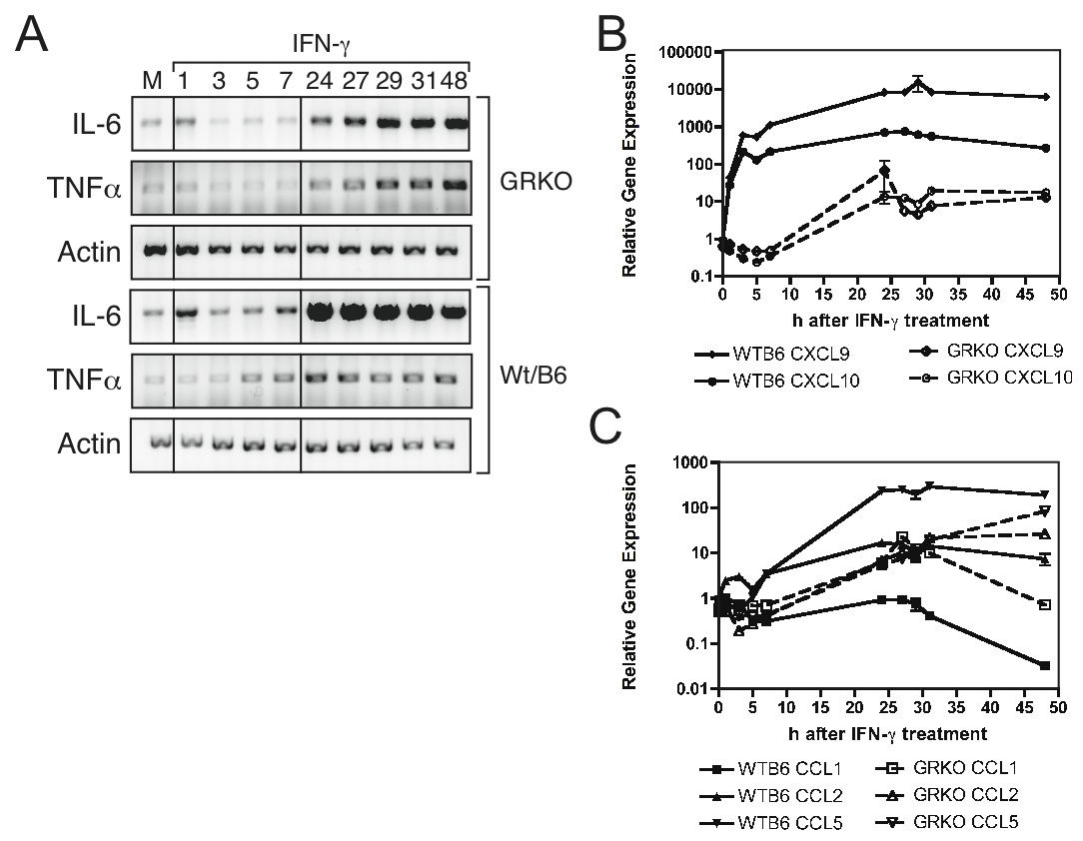
Cytokine and chemokine mRNA induction after treatment of WTB6 and GRKO MEFs with IFN-gamma. Total RNAs were collected from WTB6 and GRKO MEFs at various times after IFN-gamma treatment (100 U/mL) and assessed for levels of mRNA. (A) Changes in IL-6 and TNF-alpha mRNAs as determined by RT-PCR. Results representative of 3 experiments are shown. (B) Changes in levels of CXCL9 (diamonds) and CXCL10 (circles) mRNAs in WTB6 (solid lines) and GRKO (dashed lines) MEFs by qRT-PCR. (C) Changes in levels of CCL1 (squares), CCL2 (triangles) and CCL5 (inverted triangles) mRNAs in WTB6 (solid lines) and GRKO (dashed lines) MEFs by qRT-PCR. Data were normalized to GAPDH expression and are presented as mean +/- SEM of triplicate samples and are representative of three experiments.

 Cytokine and chemokine mRNA production was also assessed in WTB6 and GRKO MEFs treated with IFN-gamma (Figure 9). IL-6 mRNA levels were increased transiently at 1h after treatment and then again late after stimulation in both types of cells, but levels were lower in GRKO than WTB6 MEFs (Figure 9A). TNF-alpha mRNA was increased at 5h in WTB6 cells, but not until 24h after treatment in GRKO MEFs (Figure 9A). IFN-gamma rapidly induced a sustained increase in the expression of CXCL9 and CXCL10 chemokine mRNAs by WTB6 MEFs while GRKO MEFs showed a slower and lower increase (Figure 9B). There was little effect on the levels of CCL1, CCL2 or CCL5 mRNAs early after IFN-gamma treatment in GRKO MEFs or WTB6 MEFs, but there was a late increase in CCL5 mRNA expression in both types of cells (Figure 9C). Therefore, GRKO cells partially and selectively respond to IFN-gamma with production of TNF-alpha, IL-6, CCL5, CXCL9 and CXCL10 mRNAs, but responses are diminished compared to those of WTB6 cells.

### Expression and localization of IFN-gammaR2

**Figure 10:**
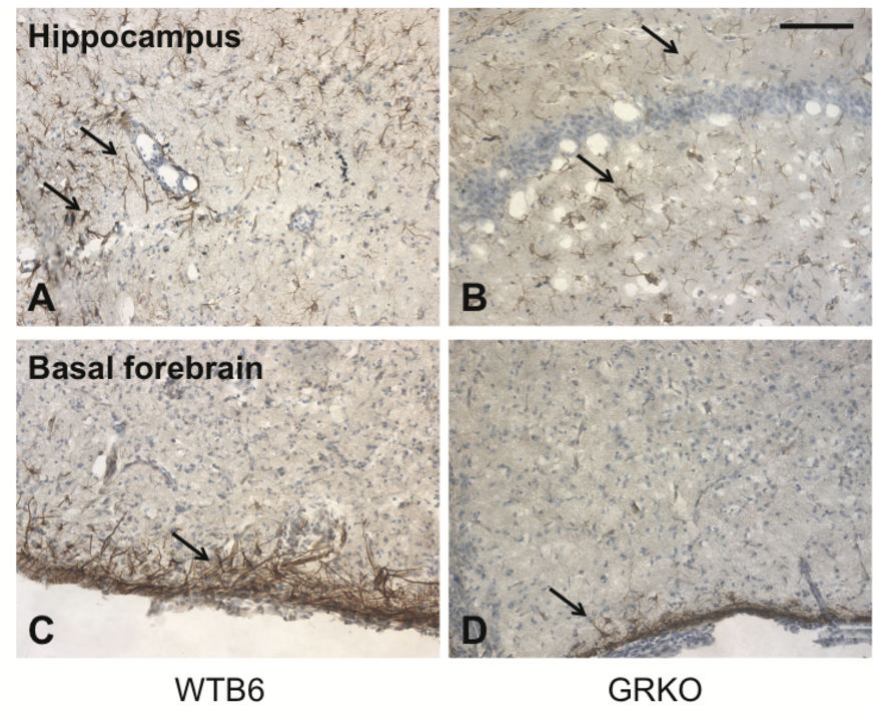
Immunohistochemical staining for IFN-gamma R2 in brains of WTB6 and GRKO mice. The hippocampus (A,B) and basal forebrain (C,D) of NSV-infected WTB6 (A,C) and GRKO (B,D) mice were examined 7 days after infection for expression of IFN-gamma R2. Frozen sections of the brains from 2 animals/group were immunostained with hamster monoclonal anti-IFN-gamma R2. IFN-gamma R2 was intensely expressed by cells in the brains of WTB6 mice (arrows). In GRKO mice that have a disruption in the IFN-gamma R1 gene, expression was less (arrows), but the distribution was similar. X200 Scale bar = 100 um.

The IFN-gamma receptor complex consists of 2 receptor subunits, IFN-gammaR1 and IFN-gammaR2, and the associated tyrosine kinases Jak1 and Jak2 [34]. Both receptor subunits are required for full biologic activity. Because expression of the IFN-gammaR2 chain is regulated while the high affinity, ligand-binding IFN-gammaR1 chain is constitutively expressed [34,35], brains were stained for IFN-gammaR2 to determine whether the lack of the transmembrane and cytoplasmic domains of IFN-gammaR1 affected IFN-gammaR2 expression in the CNS during infection (Figure 10). In the pyriform cortex at 3 days after infection, IFN-gammaR2 on astrocytes was increased and the walls of enlarged vessels were also frequently positive. At 5 days after infection, IFN-gammaR2-positive cells were increased particularly in the molecular layer of the cerebral cortex. At 7 days after infection, strong expression of IFN-gammaR2 was observed in the cerebral cortex, hippocampus and the basal forebrain (Figure 10). Patterns were similar, but GRKO mice had a lower level of expression than WTB6 mice. 

## Discussion

In viral infections, IFN-gamma has the potential for roles both in inducing an antiviral response and in regulating the immune response. Comparison of the responses of GKO and GRKO mice with the responses of WTB6 mice to infection with a neurovirulent strain of SINV revealed no differences in mortality and similar IFN-gamma-related deficiencies in the clearance of virus infection. In addition, both GRKO and GKO mice had more perforin^+^ cells infiltrating the CNS and lower expression of TNF-alpha and IL-6 mRNAs than WTB6 mice. However, there were differences in other aspects of the inflammatory response. GRKO mice had more intense inflammation and higher levels of IL-17 mRNA than WTB6 mice with an increase in the numbers of CD3^+^ T cells infiltrating the brain and greater expression of MHC-II. In contrast, GKO mice had less intense inflammation than either WTB6 or GRKO mice with fewer CD4^+^ T cells and lower expression of both MHC-I and MHC-II. Therefore, GRKO and GKO mice were comparable with respect to virus clearance, but differed in their inflammatory responses to infection.

 IFN-gamma plays an important role in clearance of virus infection from neurons. Previous studies with an avirulent strain of SINV demonstrated that in the absence of antibody, IFN-gamma was essential for clearance of SINV from the CNS [20]. In the current studies with a neurovirulent strain of SINV, clearance was compromised in both GKO and GRKO mice compared to WTB6 mice (Figures 1-3). This was evidenced by high levels of infectious virus and sustained expression of viral structural and nonstructural proteins 7 days after infection compared to WTB6 mice. An important role for IFN-gamma in clearance has also been observed for other RNA viruses [5,8,9,36–43]. 

Failure of NSV clearance was associated with an increase in infiltration of perforin^+^ cells in both GKO and GRKO mice (Figure 5). This increase may be driven by prolonged NSV replication with increased infiltration of cytotoxic cells [44] or by lack of appropriate contraction of the T cell response [45–47] as IFN-gamma has an important cell type-dependent role in regulating lymphocyte proliferation and apoptosis [48]. An increase in the local perforin^+^ CD8^+^ T cell response occurs in GKO mice in response to lymphocytic choriomeningitis virus (LCMV) infection of the CNS [36,37] while in influenza virus infection, there is an increase in CD8^+^ T cells in secondary lymphoid tissue, but not in the lungs of GKO mice [49]. Increased numbers of perforin^+^ cells are also consistent with previous studies showing that absence of IFN-gamma does not affect the cytotoxic T cell response to a variety of pathogens [50–52].

Perforin/granzyme-mediated cytolysis is an important mechanism by which NK cells and T cells eliminate virus-infected cells and this pathway can function independently, or in conjunction with IFN-gamma, for virus clearance [53–55]. Perforin^+^ CD8^+^ T cells are required for clearance of West Nile virus from infected neurons [56] and for clearance of Theilers murine encephalomyelitis virus (TMEV) and influenza virus [57,58]. However, perforin^+^ T cells are not necessary for clearance of mouse hepatitis virus [59] or for protection from Borna disease virus infection of the CNS [60]. Despite infiltration of more perforin^+^ cells, virus decrease was delayed in GKO and GRKO mice suggesting that perforin^+^ cells are insufficient for NSV clearance and that IFN-gamma is essential.

Although perforin^+^ cells were increased in both GKO and GRKO mice compared to WTB6 mice, the overall intensity of the inflammatory response was increased only in GRKO mice (Figure 5). Higher numbers of CD3^+^ T cells, suggesting augmentation of T cell infiltration into the CNS of GRKO mice, also occurs in response to TMEV infection [43]. Enhanced footpad swelling, attributed to the higher levels of virus, also occurs in GRKO mice in response to LCMV infection [52]. Levels of NSV were the same in GRKO and GKO mice (Figure 1), so higher levels of virus do not explain the increased inflammatory response in GRKO mice. Rather, the increase in IL-17 mRNA (Figure 8) suggests that Th17, and possibly Tc17, cells are preferentially expanded. IFN-gamma can suppress development of Th17 cells, but the regulatory relationship is complex [61–63]. In CNS disease, Th17 cells are effectors in some models of experimental autoimmune encephalomyelitis [64,65] and IL-17 has been implicated in both virus persistence and fatal encephalomyelitis, particularly in the absence of IFN-gamma [66–68]. It is also worth noting that GRKO mice are more susceptible to fatal herpes simplex encephalitis than GKO or WT mice without showing a difference in virus replication [69]. In our study, increased IL-17 mRNA was not associated with a difference in outcome, but the role and regulation of Th17 cells and the cytokines produced by these cells during alphavirus encephalomyelitis is worthy of further study.

Signaling through the IFN-gamma receptor can involve accessory molecules in addition to IFN-gamma R1 and IFN-gamma R2 and activation of molecules in addition to those in the classic Jak1, Jak2 and Stat-1 pathway [70,71]. Induction of an antiviral state by IFN-gamma appears to require more complex signaling than is required for induction of many other IFN-gamma-responsive genes [72,73]. In GRKO mice the IFN-gamma R1 gene is disrupted by insertion of the neomycin resistance gene into exon V, which encodes an extracellular, membrane-proximal portion of the receptor subunit leaving the ectodomain of IFN-gamma R1 intact [52]. Other defects in IFN-gamma R1 or Jak1 often lead to failure of IFN-gamma to induce an antiviral response, but allow prolonged activation of a subset of IFN-gamma-responsive genes [74]. IFN-gamma also upregulates expression of proteins, such as SOCS-1, that attenuate the IFN-gamma response [48]. An imbalance in the cellular response could lead to induction of inflammatory and MHC genes, but not antiviral or regulatory genes. A deficit in virus clearance is consistent with the lack of induction of antiviral activity in GRKO cells treated with IFN-gamma (Figure 4) [52], but the increased inflammatory responses of GRKO mice suggest that some IFN-gamma signaling occurs, perhaps through the mutated IFN-gamma R1 and intact IFN-gamma R2 or IFN-gamma R2 alone [70,75,76]. This is further suggested by the induction of IL-6 and TNF-alpha mRNAs by IFN-gamma treatment of GRKO MEFs (Figure 9).

A variety of cytokines regulate expression of MHC-I, but IFN-gamma has a particularly important role in regulation of MHC-II through induction of CIITA [48,77]. Therefore, the decrease observed in GKO mice was expected while the increase of MHC II expression in GRKO mice was not, and is further evidence that some signaling remains intact. Increased MHC-II expression has also been observed in GRKO mice in response to gentamicin toxicity in the kidney [78] and axotomy in the CNS [79]. Higher levels of MHC-II in GRKO mice than WTB6 mice (Figure 7) suggest that down regulation is not as active in mice with a mutated IFN-gammaR1.

GKO mice had lower numbers of CD4^+^ T cells and lower MHC-I and MHC-II expression than either WTB6 or GRKO mice (Figures 5-7). A decrease in the CD4^+^ T cell response also occurs in GKO mice infected with TMEV [8]. This response to viral encephalitis appears to be in contrast to what is observed in demyelinating disease [80-83]. The reasons for this difference are not clear, but suggest that GKO and GRKO mice differently modulate the CNS inflammatory response to virus-induced encephalomyelitis and to demyelinating disease.

These studies confirm a role for IFN-gamma in virus clearance. Although GKO and GRKO mice are often used interchangeably to probe the role of IFN-gamma in a variety of immunologic processes, we found differences in the number of infiltrating CD3^+^ and CD4^+^ cells and expression of MHC-I and MHC-II proteins between these strains during viral encephalomyelitis. Therefore, GRKO and GKO mice cannot be considered equivalent in their responses to viral infection.

## Materials and Methods

### Ethics statement

All animal studies were conducted in accordance with protocols approved by the Johns Hopkins University Animal Care and Use Committee (MO12H196). Mice were monitored twice a day. To assure access for paralyzed mice, food and water were provided in the cage. Ability to recover from infection is a host-dependent variable under evaluation. Because previous studies have shown that even severely paralyzed mice can survive infection and recover motor function, death was used as an endpoint. However, euthanasia was considered for animals unable to reach food or water. No animals were euthanized for this reason during the study.

### Mice

C57BL6/J (WTB6), B6·12957-IFN-gamma^tmits^ (JR2287) IFN-gamma-/- (GKO) [84], and B6·12957IFN-gammaR^tmlAgt^ (JR3288) IFN-gamma receptor1-/- (GRKO) [52] mice were purchased from the Jackson Laboratory (Bar Harbor, Maine) and used at 5-9 weeks of age. To produce primary WTB6 and GRKO MEFs, 14-day embryos were minced and trypsinized and cells were cultured in Dulbecco’s modified Eagle’s medium (DMEM) supplemented with 10% fetal bovine serum (FBS). 

### Virus and virus replication

NSV [23] was grown and assayed by plaque formation in BHK-21 cells. MEFs were infected at a multiplicity of 5 with and without 24h pretreatment with 100 U/mL recombinant rat IFN-gamma (Interferon Source). Virus replication in MEFs was assessed by titration on BHK cells of infectious virus released into the supernatant fluid. Cell viability was determined by trypan blue exclusion. 

Mice were infected intranasally with 10^5^ pfu/ml NSV in 15 uL DMEM. For assessment of mortality, mice were followed daily for signs of disease. To assess virus replication, mice were deeply anesthetized and perfused with cold PBS 3, 5 and 7 days after infection. Brains and spinal cords were removed from 3 mice in each group and stored at -80°C. Frozen tissues were weighed and homogenized with DMEM containing 1% FBS, 2mM glutamine, 100 U penicillin/ml, and 100 ug streptomycin/ml. Homogenates were clarified and assayed for infectious virus by plaque formation on BHK cells. Plaque assays were performed in triplicate. Data are presented as geometric means +/- the standard error of the mean (SEM).

### Immunohistochemistry

At least 3 animals per group were perfused with cold PBS followed by 4% paraformaldehyde. For paraffin-embedded sections, tissues were postfixed at 4°C overnight before embedding. Antigen retrieval was performed on deparaffinized sections for 20 sec with boiled sodium citrate buffer (10 mM, pH 6.0). For frozen sections, tissues were cryoprotected with 30% sucrose in PBS and 20 um sections were air-dried. Endogenous peroxidase was inactivated with ice-cold methanol containing 1% H_2_O_2_. Sections were blocked for 1h with PBS containing 1% normal goat serum and 0.4% triton X-100. 

Primary antibodies included rabbit polyclonal antibodies to SINV (1:100) [85], SINV nonstructural protein (nsP) 3 (1:200) [86], mouse CD3 (DAKO, 1:200), CD4 (Santa Cruz, 1:50) and Iba-1 (Wako, 1:100); rat monoclonal antibodies to perforin (Abcam, 1:200), CD45 (BD Pharmingen, 1:100) and B220 (BD Pharmingen, 1:50); mouse monoclonal antibodies to I-A/I-E (BD Pharmingen, 1:100) for detection of MHC class II and to H-2k^b^/H-2D^d^ (Biolegend, 1:100) for detection of MHC class I; and hamster monoclonal antibody to IFN-gamma R2 (Santa Cruz, 1:100).

Sections were incubated with primary antibody at 4°C overnight, washed with PBS containing 0.05% triton X-100 and incubated with biotinylated secondary antibody (1:100) for 1h at room temperature. Sections were then washed, incubated with ABC complex (Vectastain) followed by 0.05% diaminobenzidine (DAB) in PBS containing 0.003% H_2_O_2_ and lightly counterstained with hematoxylin. 

For quantification of immune cells expressing CD3, CD4, perforin, B220 and Iba1, frozen brain sections from 2 animals from each group were stained and 2-3 representative inflammatory foci from 3 different sections containing hippocampus were selected. Photographs of representative inflammatory foci were taken (X200, 0.25mm^2^) and immune cells per unit area (13-16 areas) were counted. For quantification of MHC-I and II-positive cells, frozen brain sections from 2 animals from each group were stained and photographs of representative inflammatory foci were taken (X250, 0.16mm^2^). For MHC-I, stained cells from 5-7 areas of thalamus were counted for each animal. For MHC-II, stained cells from 10 areas of brainstem and 5-6 areas of thalamus were counted for each mouse.

### Reverse transcriptase-polymerase chain reaction (RT-PCR) for cytokine mRNAs

At 1, 3, 5 and 7 days after intranasal infection with NSV, 3 animals per group were deeply anesthetized and perfused with cold PBS. A half brain was homogenized in 1 ml RNA STAT-60 (Tel-test, Friendswood, TX) and 0.2 ml of chloroform was added for 3 min and centrifuged at 12,000 x *g* for 15 min at 4°C. The RNA-containing aqueous phase was collected and 0.5 ml isopropanol added for 7-10 min and centrifuged at 12,000 x *g*. 1 ml 75% ethanol was added to the RNA pellet and the tubes were centrifuged at 7500 x *g*. The washed RNA pellet was air-dried and resuspended in 40 ul of DEPC-treated water containing 2 ul RNase OUT (Invitrogen).

For preparation of brain cDNA, 4-5 ug RNA were mixed with 50 ng random hexamers (Invitrogen), 1 mM dNTP mix and DEPC-treated water to a volume of 10 ul and incubated at 65°C for 5 min, then placed on ice for 1 min. Nine ul of reaction mixture (2 ul 10X RT buffer, 4 ul 25 mM MgCl_2_, 2 ul 0.1M DTT, and 1 ul RNase OUT) were added to each RNA/primer mixture and incubated at 25°C for 2 min. Finally, 1 ul SuperScript II RT was added and incubated at 25°C for 10 min. The tubes were transferred to 42°C for 50 min, 70°C for 15 min, and then chilled on ice. One ul of RNase H was added and incubated for 20 min at 37°C. A manufacturer-provided RNA-positive control was processed simultaneously. For a negative control, RT was omitted. 

Quantitative PCR amplifications were carried out with a mixture of 12.5 ul Mastermix (Applied Biosystems), 1.25 ul 20X primer and probe, 6.25 ul DEPC-treated water and 5 ul cDNA diluted 1:10. Primers and probes for cytokine mRNAs were from the TaqMan Gene Expression Assay kit (Applied Biosystems): IFN-gamma (Mm 01168134_m1), TNF-alpha (Mm 00443260_g1), IL-1alpha (Mm 00439620_m1), IL-2 (Mm 00434256_m1), IL-4 (Mm 00445259_m1), IL-6 (Mm 00446190_m1), IL-10 (Mm 00439614_m1), IL-12alpha (Mm 00434165_m1), IL-13 (Mm 00434204_m1), and IL-17a (Mm 00439619_m1). The PCR conditions were: 2 min at 50°C, 10 min at 95°C, and then 40 cycles of denaturation at 95°C for 15 sec and annealing-elongation at 60°C for 1 min. Stock cDNA pooled from all samples was used to construct standard curves. The quantities of cytokine mRNAs were normalized to the quantity of actin mRNA (Mm 00607939_s1). Two to four runs of qPCR were performed for each sample. 

For preparation of MEF cDNA, total RNA was isolated using an RNeasy kit (Qiagen). cDNA was produced with random primers using High Capacity cDNA Reverse Transcription kit (Applied Biosystems) according to the manufacturer’s instructions. PCR amplification was performed using Taq polymerase (New England Biolabs) with the following conditions: 10 min at 95°C and then 28 cycles of denaturation at 95°C for 30 sec and annealing at 55°C for 1 min and elongation at 72°C for 1 min, followed by a 5 min elongation step. The following primer sets were used: OAS - 5’GCTGATGTCAAATCAGCCGTCAA-3’, 5’-GCTCCGTGAAGCAGGTAGAGA-3’; ZAP - 5’-GCAGGTCAACTGCAACAAGAACCA-3’, 5’-AAACTGGCCTTCTCACAACATGCC-3’; TNFα - 5’-ATGAGCACAGAAAGCATGATCCGC-3’, 5’-TGAGATAGCAAATCGGCTGACGGT-3’; IL-6–5’-ATCCAGTTGCCTTCTTGGGACTGA-3’, 5’-AACGCACTAGGTTTGCCGAGTAGA-3’. PCR products were analyzed by electrophoresis using a 1% agarose gel. 

### Statistical analysis

Differences in mortality between groups were assessed by Kaplan Meier analysis with log rank (Mantel-Cox) test. Differences in other parameters between groups were assessed by Student’s unpaired t test or 1-way ANOVA (Prism 5).
